# A Cone-Beam Computed Tomographic Study on Mandibular First Molars in a Chinese Subpopulation

**DOI:** 10.1371/journal.pone.0134919

**Published:** 2015-08-04

**Authors:** Xin Zhang, Shijiang Xiong, Yue Ma, Ting Han, Xinyu Chen, Fang Wan, Yating Lu, Songhe Yan, Yan Wang

**Affiliations:** 1 VIP Center and Shandong Provincial Key Laboratory of Oral Biomedicine, School and Hospital of Stomatology, Shandong University, Jinan, Shangdong, China; 2 Department of Endodontics, School and Hospital of Stomatology, Shandong University, Jinan, Shangdong, China; University of Sheffield, UNITED KINGDOM

## Abstract

The purpose of this study was to conduct a cone-beam computed tomographic (CBCT) investigation on the root and canal configuration of the mandibular first molars, especially the morphology of the disto-lingual (DL) root, in a Chinese subpopulation. A total of 910 CBCT images of the mandibular first molars were collected from 455 patients who underwent CBCT examinations as a preoperative assessment for implants or orthodontic treatment. The following information was analyzed and evaluated: tooth position, gender, root and root canal number per tooth, root canal type of the mesial root(s) and distal root(s), angle of the DL root canal curvature, distance between two distal canal orifices in the teeth with DL root, and angle of disto-buccal canal orifice–disto-lingual canal orifice–mesio-lingual canal orifice (DB-DL-ML). Most of the mandibular first molars (64.9%, n = 591) had two roots with three root canals, and most of the mesial root canals (87.7%, n = 798) were type VI. The prevalence of the DL root was 22.1% (n = 201). The right side had a higher prevalence of DL root than the left side (*p*<0.05). Additionally, the curvature of the DL root canal were greater in the bucco-lingual (BL) orientation (30.10°±14.02°) than in the mesio-distal (MD) orientation (14.03°± 8.56°) (*p*<0.05). Overall there was a high prevalence of DL root in the mandibular first molars, and most of the DL roots were curved in different degrees. This study provided detailed information about the root canal morphology of the mandibular first molars in a Chinese subpopulation.

## Introduction

Comprehensive knowledge of root and canal morphology is fundamental for successful root canal treatment. The complexity of the root canal system determines the difficulty of root canal treatment. The omission of root canals may leave microorganisms and infectious pulp tissue untouched, which could cause post-treatment disease [1].

As the first permanent teeth appearing in our oral cavity, the mandibular and maxillary first molars are considered to be “the key of the occlusion”. The mandibular first molar is the most susceptible to caries and the most frequent to undergo root canal treatment. Studies about the root and canal morphology of mandibular first molars have never ceased [2–9]. The presence of the DL root is regarded as an important variation. A review about mandibular first molars with disto-lingual roots reported an average frequency of 14.4% of the DL root [10]. The frequency of the DL root was evidently associated with certain ethnic groups [2]. Additionally, the Chinese population was thought to have a relatively higher prevalence of the DL root in the mandibular first molars than others [2, 4, 5, 10, 11].

The presence of the DL root has brought more challenges for the root canal treatment of mandibular first molars. The DL root canal orifice can easily be overlooked, which may lead to omission of the DL canal and result in treatment failure. In addition to the challenges with its exploration, the instrumentation and obturation of this additional root have also posed challenges because the root is normally curved [12]. It is widely believed that the risk of instrument fracture significantly increases as the angle of curvature increases [13, 14].

Many kinds of methods have been used for studies on root canal morphology of mandibular first molars. So far, studies on extracted teeth with the clearing technique have provided precise observations and measurements [2, 9]. However, it is a tall order to collect large numbers of specimens; furthermore, the process is unrepeatable due to its destruction to the teeth. Periapical radiographs have generally been used in clinical examinations, but the defect of overlapping in the two-dimensional images has restricted its application in investigations [5, 15]. Recently, micro-computed tomography (micro-CT) has been used in evaluations of the tooth anatomy [16–18]. It can provide a three dimensional image without destroying the structure of the teeth. The same as the clearing technique, micro-CT can’t reflect the root and canal morphology in a living body, either. CBCT, as a three-dimensional, non-invasive tool, can directly be used in clinical examinations [19], and it is reported to be as accurate as the staining and clearing technique for evaluating root canal systems [6, 7, 20].

The purpose of this study was to conduct a CBCT investigation of the root and canal configuration of the mandibular first molars, especially the DL root configuration, in a Chinese subpopulation.

## Materials and Methods

### Sample selection

This study was conducted in the Hospital of Stomatology, Shandong University, Jinan, China. From August 2013 to July 2014, a total of 910 CBCT images of the mandibular first molars were collected from 455 patients who accepted CBCT projection as a preoperative assessment for implants or orthodontic treatment. Written informed consent was obtained from the patients, and this study was approved by the Research Ethics Committee of Shandong University Dental School. The samples were selected according to the following criteria:

1. Available CBCT images of the mandibular first molars with complete root formation
2. Absence of root canal treatment
3. Absence of coronal or post coronal restorations
4. Absence of root resorption or periapical lesions
5. CBCT images of High-quality

### Image Acquisition

The CBCT images were acquired using a CBCT scanner (Galileos, Sirona, Germany) at 85 kV and 35 mA by an experienced radiologist. The entire acquisition process was performed according to the manufacturer’s recommended protocol. The exposure time was set at 2–6 seconds, the voxel size of the images was 0.125 mm, and the slice thickness was 1.0 mm. During scanning, necessary preventive measures were taken for the patients.

### Image Evaluation

Two endodontists evaluated the images together using the inbuilt software package SIDEXIS XG 2.53. First, suitable planes were selected for our measurement and evaluation. Then, the contrast and brightness of the images were adjusted to ensure optimal visualization. All of the images from the 910 mandibular first molars were evaluated to acquire the following information:

1. Tooth position
2. Gender
3. Root number and root canal number for each tooth
4. Root canal type of the mesial root(s) (regardless of the root number) and distal root(s)
5. Presence and curvature of the DL root (in both BL and MD orientations)
6. Distance between two distal canal orifices in the tooth with the DL root.
7. Angle of DB-DL-ML.

The root canal type was classified according to the criteria of Vertucci [21]. The angle of the DL root curvature was measured according to the methods of Schneider [22].

**Fig 1:**
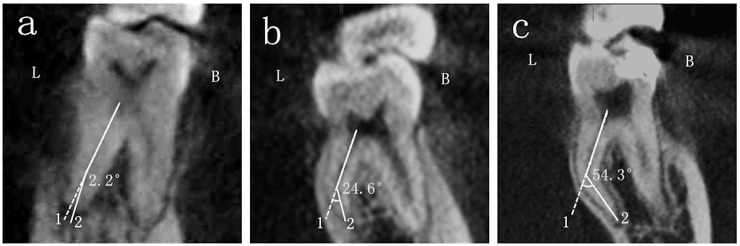
The angle of the DL root canal curvature in the BL orientation. The three pictures displayed curvatures of DL root in the BL orientation of different degrees: a, straight root canal; b, moderately curved root canal and c, severely curved root canal. L, lingual side; B, buccal side.

**Fig 2:**
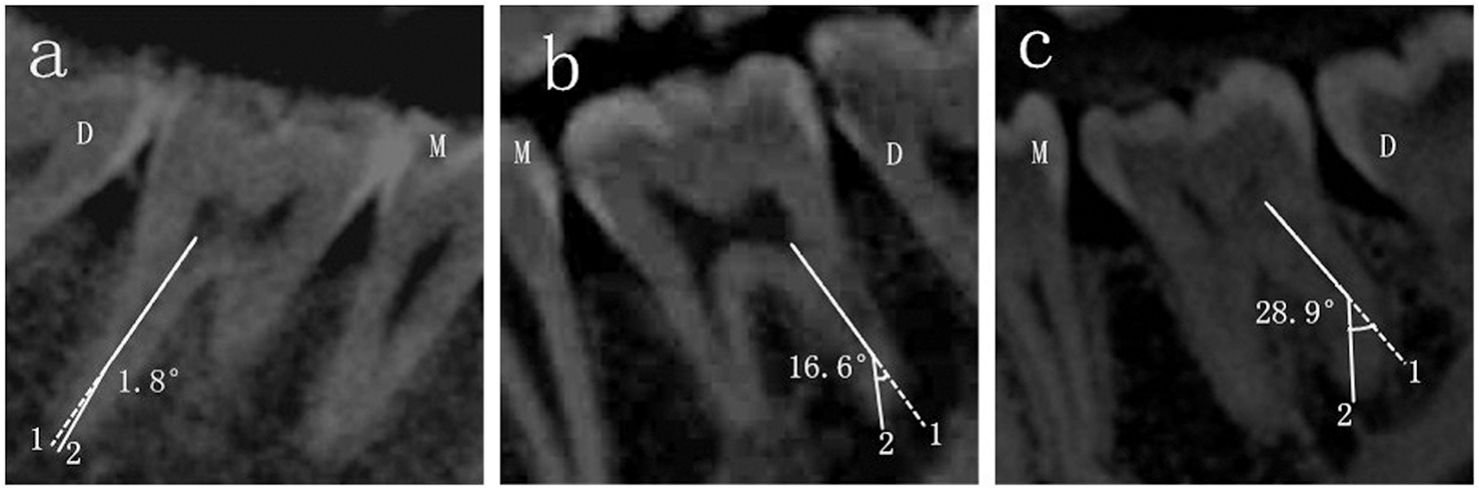
The angle of the DL root canal curvature in the MD orientation. The three pictures displayed curvatures of DL root in the MD orientation of different degrees: a, straight root canal; b, moderately curved root canal and c, severely curved root canal. M, mesial side; D, distal side.

The details about the measurement of the angle of the DL root canal curvature in the BL and MD orientations were showed in Figs 1 and 2 respectively. Choosing Panorama imaging, the curvature of the DL root canal in the BL oreintation was measured in the window of cross-sectional plane. Line 1 was the axial line of the DL root canal, line 2 connected the apical foramen and the beginning point of the canal to deviate from the axial line. The angle between line 1 and line 2 was recorded as the curvature of the DL root canal in the BL oreintation (Fig 1). The curvature in the MD oreintation was analyzed in the window of tangential plane in the same way.

**Fig 3:**
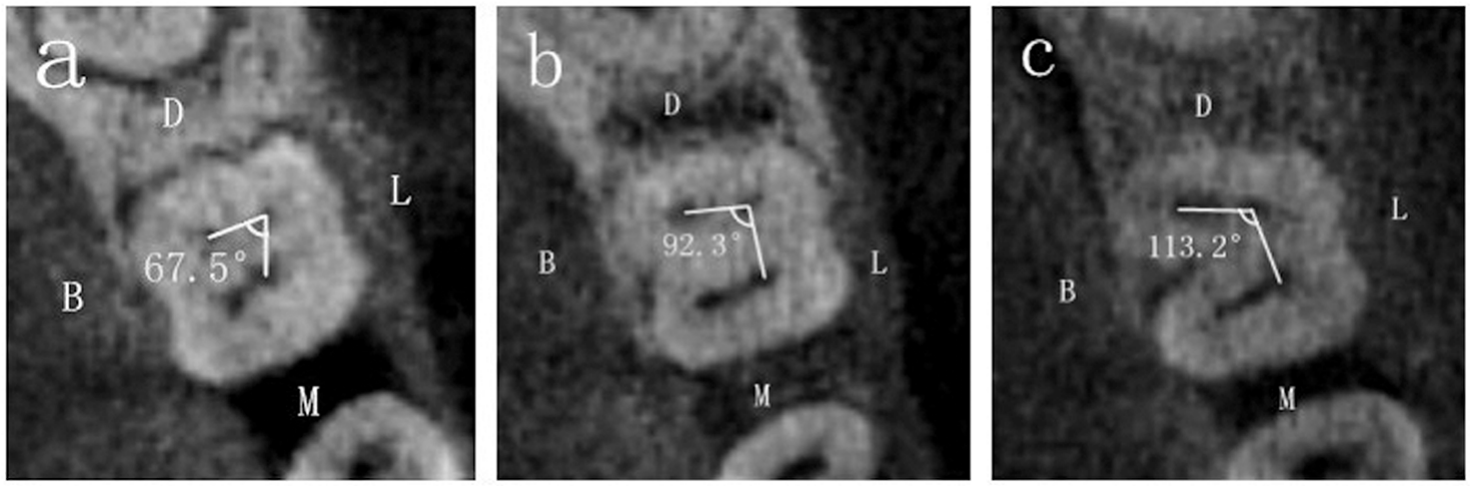
The angle of the DB-DL-ML. The three pictures showed the angles of different degrees. D, distal side; M, mesial side; L, lingual side; B, buccal side.

Fig 3 showed the measurement of the angle of DB-DL-ML. Also choosing Panorama imaging, in the window of axial (from above) plane, the center points of the DB, DL and ML were labeled in the level of pulp chamber floor.

The comparison of the angle of the DL root canal curvature between the BL and MD orientations (excluding invalid samples) was analyzed using the paired-samples t-test with SPSS 17.0 software (SPSS Inc, Chicago, IL). Differences were considered statistically significant when *p* <0.05.

The correlations between the prevalence of DL root and gender as well as tooth position were analyzed using chi-square test with SPSS 17.0 software (SPSS Inc, Chicago, IL). Differences were considered statistically significant when *p* <0.05.

## Results

One mandibular first molar had a C-shaped root morphology, which was excluded in the following evaluations.

### The number of the roots and root canals

More than half (64.9%, n = 591) of the 910 mandibular first molars had two roots with three root canals. This was the most popular type in this investigation. Three roots with four root canals were found in 21.8% (n = 198) and 109 mandibular first molars had two roots with four root canals, representing 12.0% of the sample. The details were shown in Table 1.

**Table 1:** The root and root canal number in the mandibular first molars.

	Two root canals	Three root canals	Four root canals
Two roots	5(0.5%)	591(64.9%)	109(12.0%)
Three roots	0	4(0.4%)	198(21.8%)
Four roots	0	0	2(0.2%)

### The root canal type of the mesial root(s) (regardless of the root number)

The variations in the root canal type of the mesial root(s) in this study ranged from type I to type V according to the criteria of Vertucci. Type IV was the most frequent, accounting for 87.7% of the sample (n = 798). The prevalences of type II and III were 5.6% (n = 51) and 4.8% (n = 44) respectively. Type I and V were also observed, with a similar frequency of 0.9% (n = 8). The detials were shown in Table 2.

**Table 2:** The root canal type of the mesial root(s) (regardless of the root number).

Root canal type	I	II	III	IV	V	Total
Number	8	51	44	798	8	909
Percentage	0.9%	5.6%	4.8%	87.7%	0.9%	100%

### The root canal types of distal root(s)

In the mandibular first molars with only one distal root, the variations of the root canal type ranged from type I to type IV. Most of the teeth had a single root canal (type I), accounting for 65.9% (n = 600)of the sample. Of these, 109 teeth had two distal root canals with the following distribution: type IV (9.2%, n = 84), type II (2.4%, n = 22), and type III (0.3%, n = 3). In the mandibular first molars with two distal roots, both the DB root and the DL root appeared with a single root canal (type I).

### The presence and curvature of the DL root

Among all 910 mandibular first molars, the prevalence of the DL root was 22.1% (n = 201). Of the 455 patients, 257 were male, and 198 were female. The prevalence of the DL root in the male and female patients were 23.5% (121/514) and 20.2% (80/396) respectively, with no significant difference (*p*>0.05, shown in Table 3). In addition, of all the 910 teeth, 455 teeth were from right side, with 120 DL roots (26.4%), and 455 were from left side, with 81 DL root (17.8%). The DL root preferred the right side to the left side (*p*<0.05, shown in Table 4). The angle of the DL root curvature in the bucco-lingual orientation (30.10°±14.02°) was greater than that in mesio-distal orientation (14.03°± 8.56°) (*p*<0.05). Due to the limitation of screen capturing, some teeth could not be measured, resulting in a lack of some information. The angle of the DL root curvature in the bucco-lingual orientation was evaluated in 184 teeth, most of which were classified as severely curved (the curvature of >25°) (59.8%, n = 110). Additionally, 60 teeth (33.3%) were classified as moderately curved (the curvature between 10° and 25°) and only 14 teeth (7.8%) were classified as straight (the curvature of <10°). The curvature in the mesio-distal orientation was evaluated in 199 teeth, 12.1% (n = 24) of which were severely curved, 52.3% (n = 104) were moderately curved, and 35.7% (n = 71) were straight (Table 5).

**Table 3:** The correlations between the prevalence of DL root and gender.

	Examined teeth(n)	Three roots teeth(n)
Male	514	121(23.5%)
Female	396	80(20.2%)

Level of significant was set at 0.05 (*p* = 0.229).

**Table 4:** The correlations between the prevalence of DL root and the position.

	Examined teeth(n)	Three roots teeth(n)
Right	455	120(26.4%)
Left	455	81(17.8%)

Level of significant was set at 0.05 (*p =* 0.002).

**Table 5:** The angles of the DL root canal curvature in the mandibular first molars in the BL and MD orientations.

	Straight (<10°)	Moderately curved (10°-25°)	Severely curved (>25°)	Total
BL orientation	14 (7.6%)	60 (32.6%)	110 (59.8%)	184
MD orientation	71 (35.7%)	104 (52.3%)	24 (12.1%)	199

### The distance between the two distal canal orifices in the mandibular first molars with DL roots

Most of the distances ranged from 2.5 to 3.5 mm (65.2%, n = 131). It was rare for a distance to be greater than 3.5 mm (18.9%, n = 38) or less than 2.5 mm (15.9%, n = 32).

### The angle of the DB-DL-ML

The angle of teeth ranged from 57.5°to 119.2°, and most of them were in the range of 75° to 85° (38.3%, n = 77) and 85° to 95°(35.8%, n = 72). More details were shown in Table 6.

**Table 6:** The angle of DB-DL-ML in the mandibular first molars with the DL root.

Angle	<75°	75°-85°	85°-95°	95°-105°	>105°	Total
Number	34	77	72	13	5	201
Percentage	16.9%	38.3%	35.8%	6.5%	2.5%	100%

## Discussion

This study investigated the root canal morphology of mandibular first molars, especially the DL root in a Chinese subpopulation, using CBCT. A total of 910 CBCT images of mandibular first molars from 455 patients were evaluated; of which, 201 DL roots were investigated.

The occurrence of the DL root was thought to be a major anatomical variant in the mandibular first molars, ethnic and geographical factor played an important role in this variation. In the present study from a Chinese subpopulation, the prevalence of the DL root was 22.1%. The result was significantly higher than the average prevalence of 14.4% in previous studies [10], especially the studies from African population (3.1%) [23], Eurasian population(<5%) [24] and Caucasians (3.4%-4.2%) [24, 25]. Compared with other investigations from Mongoloid populations, the result of this study was in accordance with their findings [4, 7, 8, 11, 26–28], or even lower than some studies in Chinese [6, 29] or Korean populations [30, 31]. This study further confirmed that the population from Mongoloid origin was more likely to have a DL root in the mandibular first molars.

The same as the previous studies detecting gender predilection for the DL root in the mandibular first molars, in the present study, the prevalence of DL root had no significant difference between male (121/514, 23.5%) and female (80/396, 20.2%) (*p>*0.05). Interestingly, about the detection of topologic predilection, some studies showed that the DL root was more frequent on the right side than on the left side, which was in accordance with the present study (right side, 120/455, 26.4%; left side, 81/455, 17.8%, *p<*0.05), whereas some studies were just the opposite and some other studies showed no significant difference bilaterally. Ethnic origin, sample size and methods used might play an important role in these contradictory findings.

It was reported that the entrance of the DL root canal was located disto- to mesio-lingually from the disto-buccal root canal. In our study, the angle of the DB-DL-ML in the mandibular first molars with the DL root was measured, and most of the angles were around or lower than 90°, indicating that the DL root canal was more likely to be more disto-lingual from the disto-buccal root canal. At the same time, the measurement of the distance between two distal canal orifices in the mandibular first molars with DL roots showed that most teeth had a distance of 2.5–3.5 mm. All of these findings indicated that a more trapezoidal cavity could help clinicians locate the DL root canal orifice [32].

In this study, all of these DL roots had a single root canal from the orifice to the apical foramen. Usually, the DL root canal existed with curvature of different degrees. The angle of the DL root curvature in the bucco-lingual orientation (30.10°±14.02°) was greater than that in the mesio-distal orientation (14.03°± 8.56°) (*p*<0.05), which was in accordance with previous reports. According to the classification of Schneider, more than half of the DL root canals were classified as severely curved (>25°) in the BL orientation and moderately curved in the MD orientation [12, 32]. It must be noted that, in previous studies, the angle of the DL root canal curvature was measured on radiographs of extracted teeth. However, in CBCT images, it is difficult to capture a perfectly appropriate plane for the measurement. The angles obtained were smaller than the actual planes in most cases. In addition, we had a high percentage of missed curvatures, especially in the BL orientation. In clinical practice, the curvature of the root canal could increase the difficulty in negotiating and preparing the root canal and might translate into a high incidence rate of instrument fracture. Thus, cares should be taken to avoid instrument fracture and perforation during negotiating root canals.

CBCT is a useful tool in both clinical examinations and morphological research. It can provide three-dimensional images in axial, sagittal and coronal sections, avoiding geometric distortion and anatomical superimposition in traditional radiographs. At the same time, as a non-invasive tool, CBCT is reported as accurate as the staining and clearing technique for evaluating root canal systems [20].

The study provided detailed information about the morphology of the mandibular first molars as a complement to previous studies. It could help clinicians locate the DL root canal orifice. With respect to the curvature of the DL root canals, CBCT could not provide as accurate a measurement as in vitro studies. However, the large samples gathered by CBCT images were irreplaceable, and they still had substantial reference value.
